# Exploration of early social behaviors and social styles in relation to individual characteristics in suckling piglets

**DOI:** 10.1038/s41598-022-06354-w

**Published:** 2022-02-10

**Authors:** C. Clouard, R. Resmond, A. Prunier, C. Tallet, E. Merlot

**Affiliations:** PEGASE, INRAE, Institut Agro, 35590 Saint-Gilles, France

**Keywords:** Behavioural methods, Animal behaviour

## Abstract

Social behavior is a key component of pig welfare on farms, but little is known on the development of social behaviors in piglets. This study aimed to explore social behaviors and identify early social styles in suckling piglets. Social behaviors of 68 piglets from 12 litters were scored continuously for 8 h per day at 21 and 42 days of age, and were included in a Hierarchical Clustering on Principal Components analysis to identify clusters of pigs with similar social styles. Social nosing represented 78% of all social interactions given. Three social styles were identified: low-solicited inactive animals (inactive), active animals (active), and highly-solicited avoiders (avoiders). Belonging to a cluster was independent of age, but was influenced by sex, with females being more represented in the ‘inactive’ cluster, and males in the ‘active’ cluster, whereas both sexes were equally represented in the ‘avoider’ cluster. Stability of piglets’ allocation to specific clusters over age was high in the ‘inactive’ (59%) and ‘active’ (65%) clusters, but low in the ‘avoider’ cluster (7%). Haptoglobin and growth rate were higher in ‘active’ than ‘inactive’ pigs, and intermediate in ‘avoiders’. Our findings suggest the existence of transient social styles in piglets, likely reflective of sexual dimorphism or health status.

## Introduction

The pig (*Sus scrofa*) is a highly gregarious species, which lives in highly stable social groups^1,2^ resting on a linear hierarchy based on dominant-subordinate relationships between group members^3,4^. These dominant-subordinate relationships and agonistic behaviors have received extensive research attention, whereas the relevance of socio-positive behaviors for pig welfare on farms remains poorly understood^2,5,6^. Yet, in stable groups of pigs, the incidence of agonistic interactions is low compared to that of positive social behaviors, such as ‘social nosing’^7–9^, and recent research suggests that (putative) positive social behaviors, also sometimes referred to as ‘affiliative’ or ‘prosocial’ behaviors, may be key components for welfare of animals on farms^5^. For instance, social nosing, a common contact behavior in pigs comparable to the licking and grooming behavior described in other farm species^2^ is assumed to contribute to individual recognition^10–12^, to participate in the maintenance of social relationships and group cohesion^2,6,13^, and to generate positive affective states^11,14^. Social play is another putative positive social behavior that is generally assumed to be an indicator of welfare in juvenile pigs^15–17^. Indeed, social play helps piglets to develop social skills necessary for conflict resolution and individual recognition^18,19^, and allows littermates to develop social bonds that remain quite strong even after regrouping with other litters, thus participating in group cohesion^2,10^.

Furthermore, while previous work has focused heavily on dominance relationships and agonistic behaviors, dominance rank cannot fully explain the large individual differences in social behavior and physiological reactions to social situations often shown by animals^20,21^. These individual differences in behavior and physiology have been explained by personality traits, also sometimes referred to as individual ‘strategies’ or ‘styles’, that can be defined as “a coherent set of strategies, as well as behavioral and morphological adaptations, which are consistent over time and contexts”^22^. The study of personality traits is a major challenge for animal welfare research because it may help to understand the consistent differences in behavioral and physiological responses of farm animals to various challenges and conditions, such as different housing systems, management practices or veterinary interventions^22^. In gregarious animals, sociability and aggressiveness are among the most commonly measured personality traits^22,23^. In this context, recent behavioral research has explored social personality traits, or individual social strategies or styles, in a variety of farm animals, including dairy calves^24^, cows^25^, goats^26,27^, ewes^28^, and pigs^20,29,30^. These social styles have been found to be associated with a variety of individual features. For instance, avoider, affiliative, aggressive and pragmatic ewes differed in their body length, body weight and/or thorax circumference^26–28^, while aggressive and non-aggressive or affiliative animals showed contrasted cortisol levels in ewes^28^, goats^27^ and cows^25^. Research, however, has focused almost exclusively on adult individuals, and little is known on the development of early social styles in farm animals. Yet, identifying early individual social styles integrating both (putative) affiliative and agonistic behaviors may help to predict the adaptability of animals to various challenges or husbandry practices they may encounter later in life, such as social rearrangements^5^. A better understanding of early social behavior may also help to identify putative positive social behaviors important for welfare. For instance, some social behaviors, such as rough-and-tumble play or ‘playfight’, are assumed to help piglet developing the motor and social skills needed for successful fighting^18,19,31^, thus favoring optimal integration into future hierarchical social groups^18^.

Our study aimed first to describe the social behaviors of 3- to 6-week-old suckling piglets raised in an enriched environment, and explore the existence of early social styles throughout the suckling period based on these social interactions. Next, we aimed to assess the stability of these social styles with age, and the association between these social styles and a variety of individual features, including non-social behaviors (time spent lying, standing or eating) and morphological traits (sex, weight, growth). Because health status can influence behavior, including social behaviors^32^, we also investigated the association between these social styles and health-related blood parameters (markers of inflammation: haptoglobin^33^, and markers of oxidative stress: hydrogen peroxide and plasma antioxidant capacity). Furthermore, because peripheral (and central) serotonin (5-HT) levels have been shown to be related to affiliative behaviors (for reviews, see^34,35^) and aggressive behaviors^36,37^ in a variety of species, including dogs, pigs and humans, the association between blood 5-HT levels and social behaviors and styles at both ages was explored^38^.

## Results

### Descriptive analysis of social (and non-social) behaviors

We collected a total of 3795 (emitted and received) social interactions from 68 piglets from 12 litters at 21 of age, and 6630 (emitted and received) interactions from 69 piglets from 12 litters at day 42 of age. In total, 78% of all behaviors emitted (social nosing, agonistic and other social behaviors) were social nosing (nosing nose, head and body) at both ages, while 8 and 12% were agonistic behaviors, and 14% and 9% were other social behaviors (mounting and nudging) at 21 and 42 days of age, respectively. Social play, on the other hand, represented 22% of all (emitted and received) social interactions at both ages.

**Figure 1:**
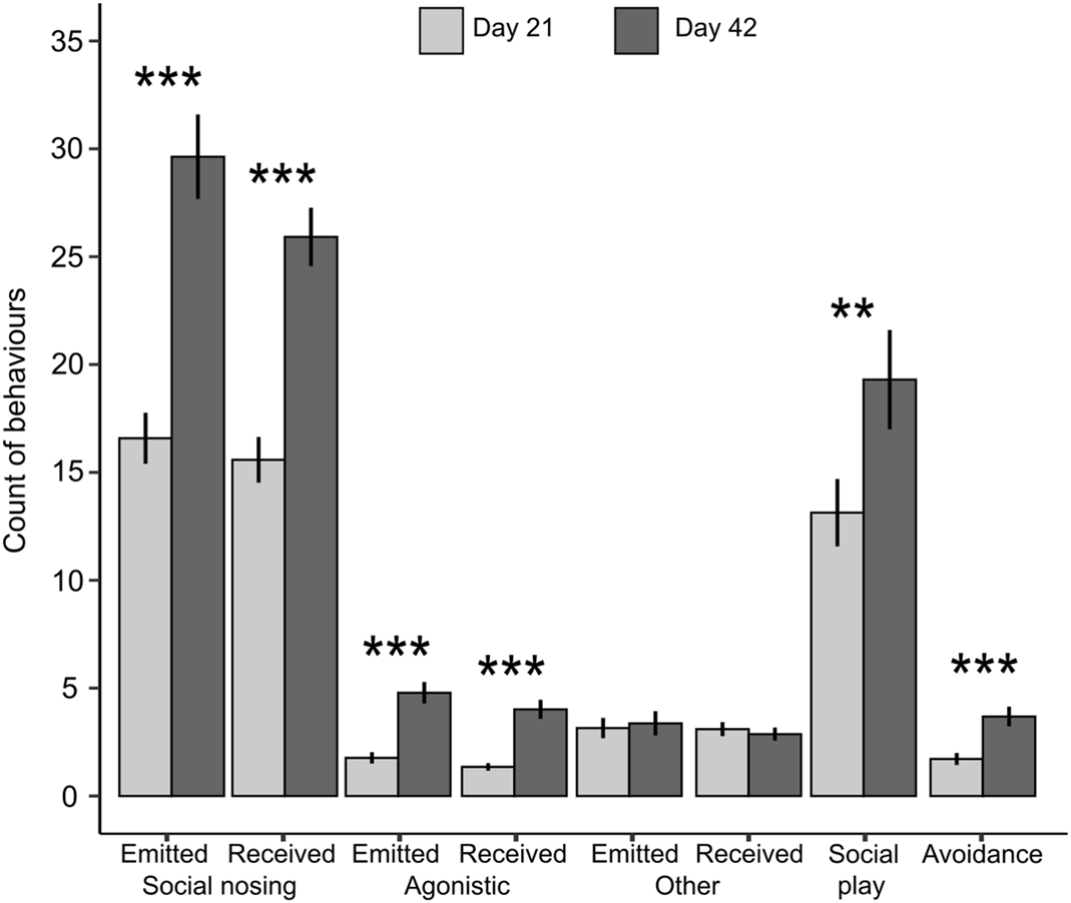
Effects of age on social behaviors given and received by pre-weaned piglets at 21 and 42 days of age. Data are presented as means ± SEM.

**Figure 2:**
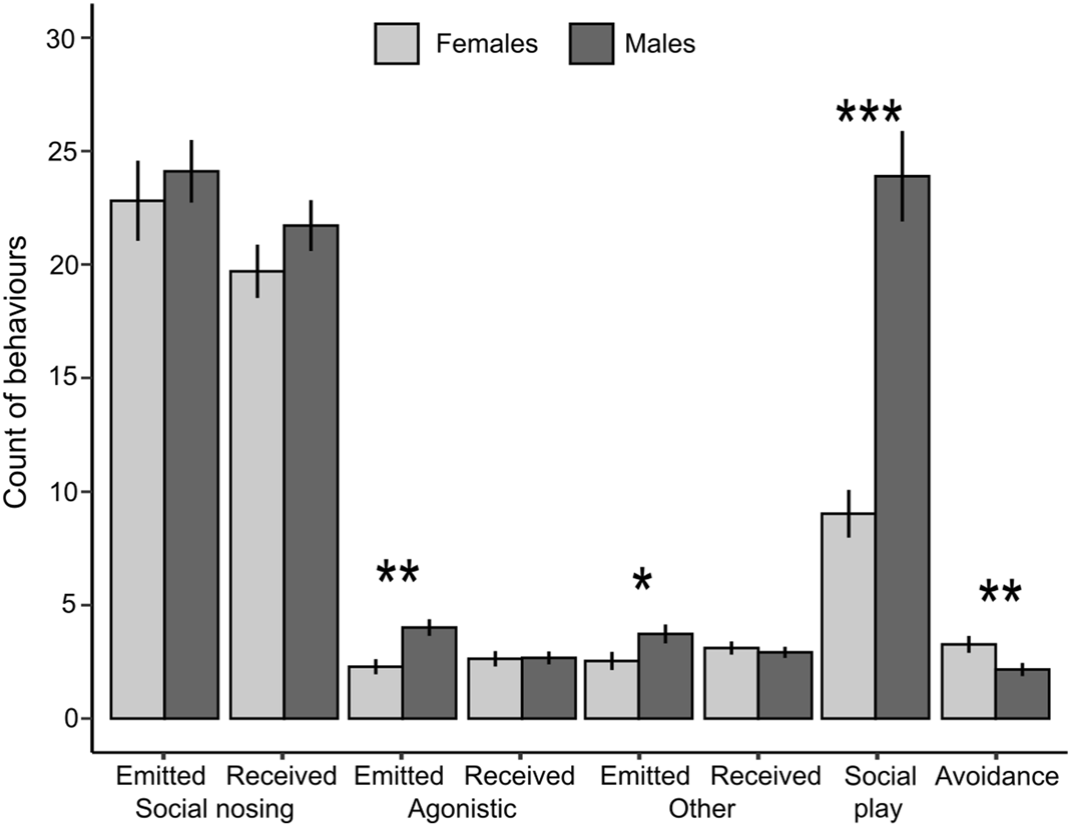
Effects of sex on social behaviors given and received by pre-weaned piglets at 21 and 42 days of age. Data are presented as means ± SEM.

Piglets were involved in more social interactions at 42 (63.4 ± 3.9 interactions/pig [range: 4–149]) than at 21 days of age (35.7 ± 2.6 [1–83], F(1,66) = 52.6, *p* < 0.001). Notably, piglets emitted more agonistic and social nosing behaviors, received more agonistic behaviors, and showed more avoidance and social play episodes at 42 than at 21 days of age (Fig. 1). Males were involved in more social interactions than females (males: 57.9 ± 3.3 interactions/pig; females: 39.9 ± 3.0; F(1,58) = 14.7, *p* < 0.001). Notably, males emitted more agonistic and other social behaviors (*i.e.* nudging and mounting), were involved in more social play episodes, but showed fewer avoidance behaviors than females (Fig. 2).

When considering non-social behaviors, piglets spent less time lying (proportion of total observations, d21: 0.58 ± 0.01; d42: 0.49 ± 0.01; F(1,72) = 24.9, *p* < 0.001), and more time standing (d21: 0.22 ± 0.008; d42: 0.34 ± 0.01; F(1,73) = 84.4, *p* < 0.001) at 42 than at 21 days of age. No effect of age was found on any other behavioral activities (time spent suckling, eating or drinking; *p* > 0.10 for all). No effect of sex was found on non-social behavioral activities, including the proportion of observations spent lying, standing, ingesting and suckling.

### Identification of individual social styles

#### Principal components analysis (PCA)

A total of 3 PC explaining 58% of the total variance were extracted from the PCA performed on the 60 piglets observed on both days (Table 1). The first PC accounted for 25% of the total variance. The number of social nosing given (loading: 0.70), other social behaviors given (0.75), and social play episodes (0.66) loaded positively on the component, which was thus labelled ‘*social exploration & social play*’. The second PC accounted for 18% of the total variance. The number of avoidance behaviors (0.77) and other received social behaviors (0.66) loaded positively, while the number of agonistic behaviors given (− 0.54) loaded negatively on the second component, which was thus labelled ‘*avoidance & receipt of exploration* vs *aggression*’. The third PC accounted for 15% of the total variance. The number of received social nosing behaviors (0.77) loaded positively on the third component, which was thus labelled ‘*receipt of social nosing*’.

**Table 1:** Coordinates of the active variables on the dimensions of the principal component analysis obtained from social behaviors at 21 and 42 days of age^a^.

	Dimension 1 ‘Social exploration & social play’	Dimension 2 ‘Avoidance & receipt of social exploration *vs* aggression’	Dimension 3 ‘Receipt of social nosing’
Variance explained	25%	18%	15%
Agonistic behaviors given	0.39	**− 0.54**	0.48
Social nosing behaviors given	**0.70**	**− **0.10	**− **0.32
Other social behaviors given	**0.75**	**− **0.04	0.04
Agonistic behaviors received	0.43	**− **0.03	**− **0.38
Social nosing behaviors received	0.21	0.31	**0.77**
Other social behaviors received	0.29	**0.66**	0.26
Social play	**0.66**	**− **0.23	0.09
Avoidance	0.29	**0.77**	**− **0.23

^a^High to moderate (> 0.50 or < − 0.50) coordinates are in bold.

#### Hierarchical clustering analysis on principal components (HCPC)

**Figure 3:**
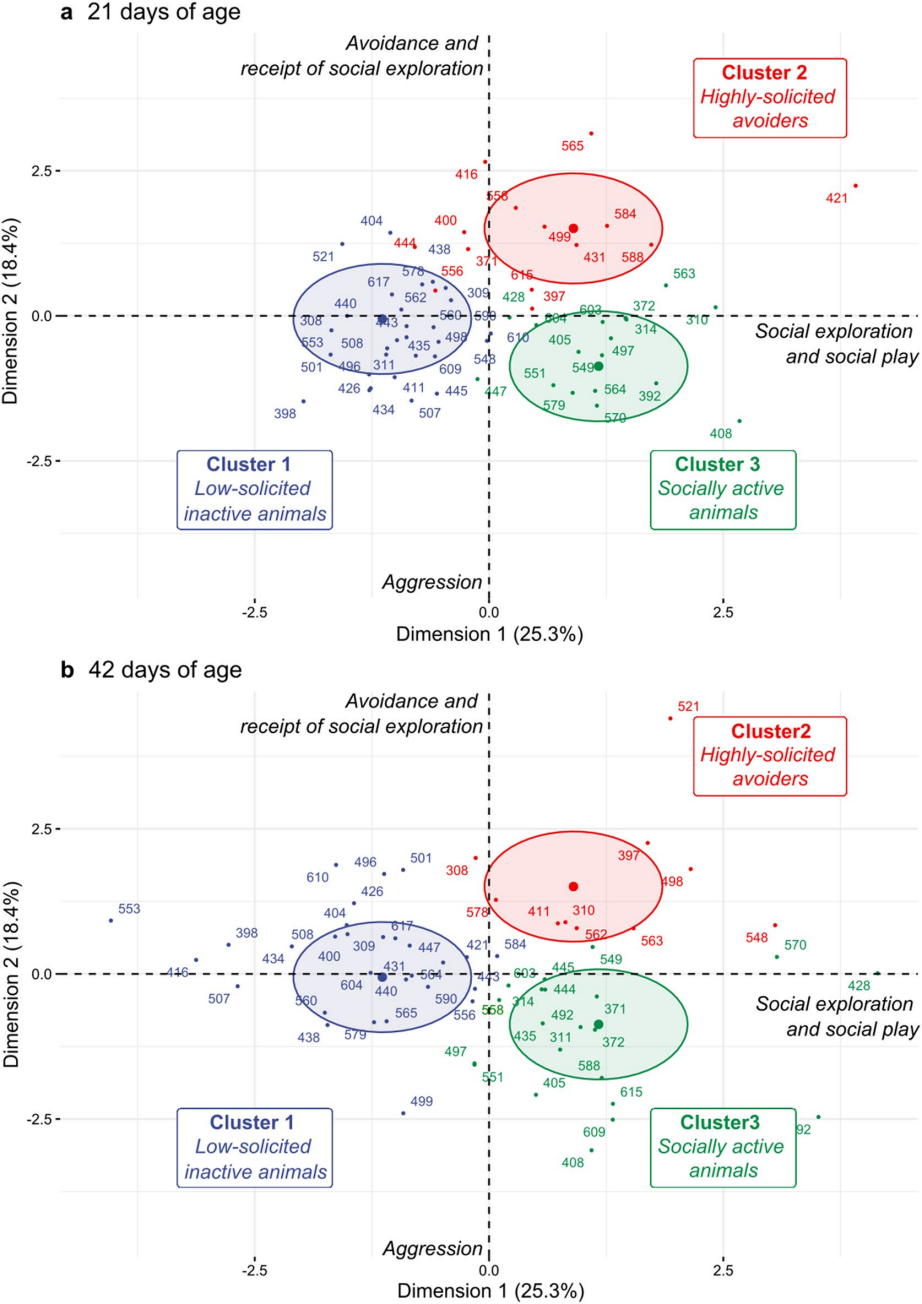
Three clustered groups of piglets differing in their social behaviors (**a**) at 21 days of age and (**b**) at 42 days of age, according to social exploration and social play (dimension 1) and to avoidance and receipt of social exploration *vs* aggression (dimension 2). Dimensions were extracted from a global Principal Component Analysis (PCA) done on the social behaviors of 60 piglets observed on both days (*i.e.* 120 observations in total). Hierarchical Clustering on Principal Components was then performed on the dimensions extracted from the PCA to identify clustered groups of pigs differing in their social behaviors. Active and supplementary variables characterizing each cluster are defined in Table 2. Ellipses of the clusters are plotted according to the Euclidian distance.

The hierarchical clustering analysis was performed on the 3 extracted PC to identify clustered groups of pigs based on social behaviors at both ages. The HCPC suggested the existence of 3 social clusters (Table **2** and Fig. 3a,b). Of the 120 observations included in the PCA (*i.e.* 60 piglets at both ages), 58 were in cluster 1 (29 and 29 piglets observed at 21 and 42 days, respectively; 48% of observations), 24 piglets in cluster 2 (14 and 10 piglets observed at 21 and 42 days, respectively; 20% of the observations), and 38 piglets in cluster 3 (17 and 21 piglets observed at 21 and 42 days, respectively; 32% of the observations). Clusters were not significantly associated with the supplemental qualitative variable ‘day’, suggesting that the distribution of piglets observed at 21 or 42 days of age was balanced between clusters (χ^2^ test, *p* > 0.10; Fig. 3a,b).

**Table 2:** Results of the hierarchical clustering analysis performed on the principal components extracted from a global principal component analysis based on social behaviors at 21 and 42 days of age.

Principal component and variables	Coordinate^a^	Cluster 1 ‘Low-solicited inactive animals’	Cluster 2 ‘Highly-solicited avoiders’	Cluster 3 ‘Socially active animals’
		*n* = 58 (d21: 29; d42: 29)	*n* = 24 (d21: 14; d42: 10)	*n* = 38 (d21: 17; d42: 21)
		*v*.test^b^	*v*.test^b^	*v*.test^b^
Principal component 1	–	− 8.45***	3.45***	6.11***
Agonistic given	0.39	–	–	–
Social nosing given	**0.70**	− 5.26***	–	4.96***
Other social given	**0.75**	− 5.62***	–	5.14***
Agonistic received	0.43	− 3.72***	–	2.35*
Social nosing received	0.21	−	–	–
Other social received	0.29	−	–	–
Social play	**0.66**	− 5.70***	–	5.16***
Avoidance	0.29	−	–	−
Total (supplementary quantitative)	**0.92**	− 7.52***	–	6.69***
Principal component 2	–	–	6.76***	− 5.31***
Agonistic given	**− 0.54**	− 4.01***	–	5.16***
Social nosing given	**− **0.10	–	–	–
Other social given	− 0.04	–	–	–
Agonistic received	− 0.03		–	–
Social nosing received	0.31	–	–	–
Other social received	**− 0.66**	− 3.80***	6.94***	–
Social play	**− **0.23	–	–	–
Avoidance	**0.77**	− 2.17*	4.87***	–
Total (supplementary quantitative)	− 0.17	–	–	–
Principal component 3	–	–	2.70**	
Agonistic given	0.48	–	–	
Social nosing given	**− **0.32	–	–	–
Other social given	0.04	–	–	–
Agonistic received	− 0.38	–	–	–
Social nosing received	**0.77**	− 2.85**	5.11***	–
Other social received	0.26	–	–	–
Social play	0.09	–	–	–
Avoidance	− 0.23	–	–	–
Total (supplementary quantitative)	**− 0.08**	–	–	

The table presents coordinates and characterizes clusters with active and supplementary variables.

^a^Coordinates (loadings) of the variable on the principal component. Variables with high coordinates on the principal component (> 0.50 or < − 0.50, in bold) are positively and negatively correlated to the principal component, respectively;

^b^Only significant *v*.test values (*v.*test > 1.96 or *v*.test < − 1.96; * *p *value < 0.05, ** *p *value < 0.01, *** *p *value < 0.001) are shown. Test values for variables are shown within the principal component section for which the variable had the highest coordinate. Principal components and variables with significant *v.*test values described the clusters obtained from the hierarchical clustering analysis. The sign of the *v.*test value indicates if the mean of the cluster is significantly lower or greater than the overall mean of all clusters.

Compared to all piglets, animals from cluster 1 had lower coordinates on PC 1 (‘social exploration & social play’, *p* < 0.001), and were characterized by fewer social play episodes, social nosing, agonistic behaviors, and other social behaviors given (*p* < 0.01 for all). Animals in cluster 1 also received fewer agonistic behaviors, social nosing and other social behaviors (*p* = 0.01), and exhibited fewer avoidance behaviors (*p* = 0.03). Overall, animals from cluster 1 were involved in fewer social interactions compared to all piglets (*p* < 0.001), and were thus labelled *‘low-solicited inactive animals*’.

Compared to all piglets, animals from cluster 2 had higher coordinates on PC 2 (‘avoidance & receipt of exploration *vs* aggression’, *p* < 0.001), PC 1 (‘social exploration & social play’, *p* < 0.001) and, to a lesser extent, PC 3 (‘receipt of social nosing’, *p* = 0.007) Cluster 2 was characterized by more social nosing and other social behaviors received (*p* < 0.001 for both), and more avoidance behaviors (*p* < 0.001). Therefore, animals from cluster 2 were labelled ‘*highly-solicited avoiders’*.

Compared to all piglets, animals from cluster 3 had higher coordinates on PC 1 (‘social exploration & social play’, *p* < 0.001) and lower coordinates on PC 2 (‘avoidance & receipt of exploration *vs* aggression’, *p* < 0.001). Cluster 3 was characterized by more social play episodes, and social nosing, agonistic behaviors and other social behaviors given (*p* < 0.001 for all), and by more agonistic behaviors received (*p* = 0.02). Overall, animals from cluster 3 were also involved in more social interactions (*p* < 0.001) than all other piglets, and were thus labelled *‘socially active animals’*.

### Characterization of social clusters with individual characteristics

Analyses of the association between the clusters and supplementary qualitative variables revealed that clusters were significantly associated with sex (χ^2^ test, *p* = 0.003). A total of 31 males and 29 females on both days were included in the multivariate analyses, that is 52% are males and 48% are females. The cluster of ‘*low-solicited inactive animals’* was typically composed of females (*v*-test = 2.88, *p* = 0.004), with 62% of pigs in this cluster being females (22 males *vs* 36 females). On the contrary, 76% of animals in the cluster of ‘*socially active animals’* were males (*v*-test = 3.67, *p* < 0.001, 29 males *vs* 9 females). In the cluster of ‘*highly-solicited avoiders’*, the sex ratio was balanced (11 males *vs* 13 females).

Effects of clusters, and their interaction with sex, on quantitative variables are presented in Table 3. Significant cluster × sex interaction effects were found for non-social behaviors . Indeed, females in the ‘*low-solicited inactive’* cluster spent more time lying than females from the *‘socially active’* cluster (post-hoc, *p* = 0.02) and less time standing than females from the ‘*socially active’* (*p* < 0.001) and ‘*highly-solicited avoiders*’ clusters (*p* = 0.002), whereas no differences were found for males. Other non-social behaviors, including time spent eating, drinking, or suckling, were not influenced by cluster, sex or their interactions (*p* > 0.05). Irrespective of sex, weight and averaged daily gain (ADG) were associated with cluster. Indeed, ‘*low-solicited inactive animals’* were lighter (7.3 ± 2.9 kg *vs* 9.0 ± 2.9 kg, *p* < 0.001) and gained fewer weight (209 ± 48.8 g/d *vs* 263 ± 49.8 g/d, *p* = 0.006) than ‘*socially active animals*’. ‘*Low-solicited inactive animals*’ also had higher haptoglobin levels (2.35 ± 0.75 mg/mL *vs* 1.32 ± 0.78 mg/mL, *p* = 0.02) than ‘*socially active animals*’. Other blood parameters, including platelet-rich plasma serotonin (5-HT), and markers of oxidative stress (FRAP and dROM), were not influenced by cluster, sex or their interactions (*p* > 0.05 for all).

**Table 3:** Behavioral, morphological and physiological characteristics of clusters obtained from a hierarchical clustering analysis on principal components based on social behaviors measured at 21 and 42 days of age^1^.

*n*	Females			Males			*p *values^5^		
	Cluster 1 ‘Inactive’	Cluster 2 ‘Avoiders’	Cluster 3 ‘Active’	Cluster 1 ‘Inactive’	Cluster 2 ‘Avoiders’	Cluster 3 ‘Active’	Cluster	Sex	Cluster × Sex
	36	13	9	22	11	29			
**Behavioral traits**^2^									
Time lying	0.58 ± 0.03^a^	0.50 ± 0.04^ab^	0.45 ± 0.04^b^	0.54 ± 0.03^ab^	0.58 ± 0.04^ab^	0.54 ± 0.03^ab^	0.09	0.08	**0.03**
Time standing	0.23 ± 0.06^a^	0.32 ± 0.06^b^	0.36 ± 0.06^b^	0.25 ± 0.06^ab^	0.25 ± 0.06^ab^	0.30 ± 0.06^ab^	** < 0.001**	**0.02**	**0.02**
Time ingesting	0.02 ± 0.003	0.02 ± 0.004	0.02 ± 0.005	0.01 ± 0.004	0.01 ± 0.005	0.02 ± 0.003	0.13	0.11	0.79
Time suckling	0.15 ± 0.02	0.14 ± 0.02	0.12 ± 0.03	0.15 ± 0.02	0.15 ± 0.03	0.17 ± 0.02	0.77	**0.02**	0.10
**Production traits**									
Weight (kg)	7.2 ± 2.9	7.6 ± 3.0	9.6 ± 3.0	7.5 ± 2.9	8.5 ± 3.0	8.4 ± 2.9	** < 0.001**	0.95	0.08
ADG^3^ (g/d)	211 ± 49	237 ± 52	276 ± 54	207 ± 50	247 ± 53	251 ± 50	**0.006**	0.66	0.69
**Blood parameters**									
5-HT (µmol/L PRP^4^)	14.0 ± 4.5	14.8 ± 4.9	11.7 ± 5.2	18.6 ± 4.7	15.2 ± 5.0	15.3 ± 4.6	0.35	0.10	0.60
Haptoglobin (mg/mL)	2.3 ± 0.77	1.2 ± 0.84	1.4 ± 0.90	2.4 ± 0.80	2.5 ± 0.86	1.2 ± 0.78	**0.02**	0.22	0.19
dROM (CARRU)	914 ± 43	842 ± 61	796 ± 74	902 ± 51	995 ± 66	868 ± 47	0.22	0.09	0.24
FRAP (µg/mL)	67 ± 3.6	69 ± 4.2	68 ± 4.7	66 ± 3.9	67 ± 4.5	68 ± 3.7	0.69	0.69	0.84

^1^LS-Means ± S.E.M.;

^2^Proportion of total observation time;

^3^ADG, average daily gain;

^4^PRP, platelet-rich plasma;

^5^Letters indicate differences between groups as estimated by post-hoc test with Tukey adjustment for comparing multiple groups (*p* < 0.05).

### Stability of social behaviors and social styles over time

#### Consistency of social behaviors

**Figure 4:**
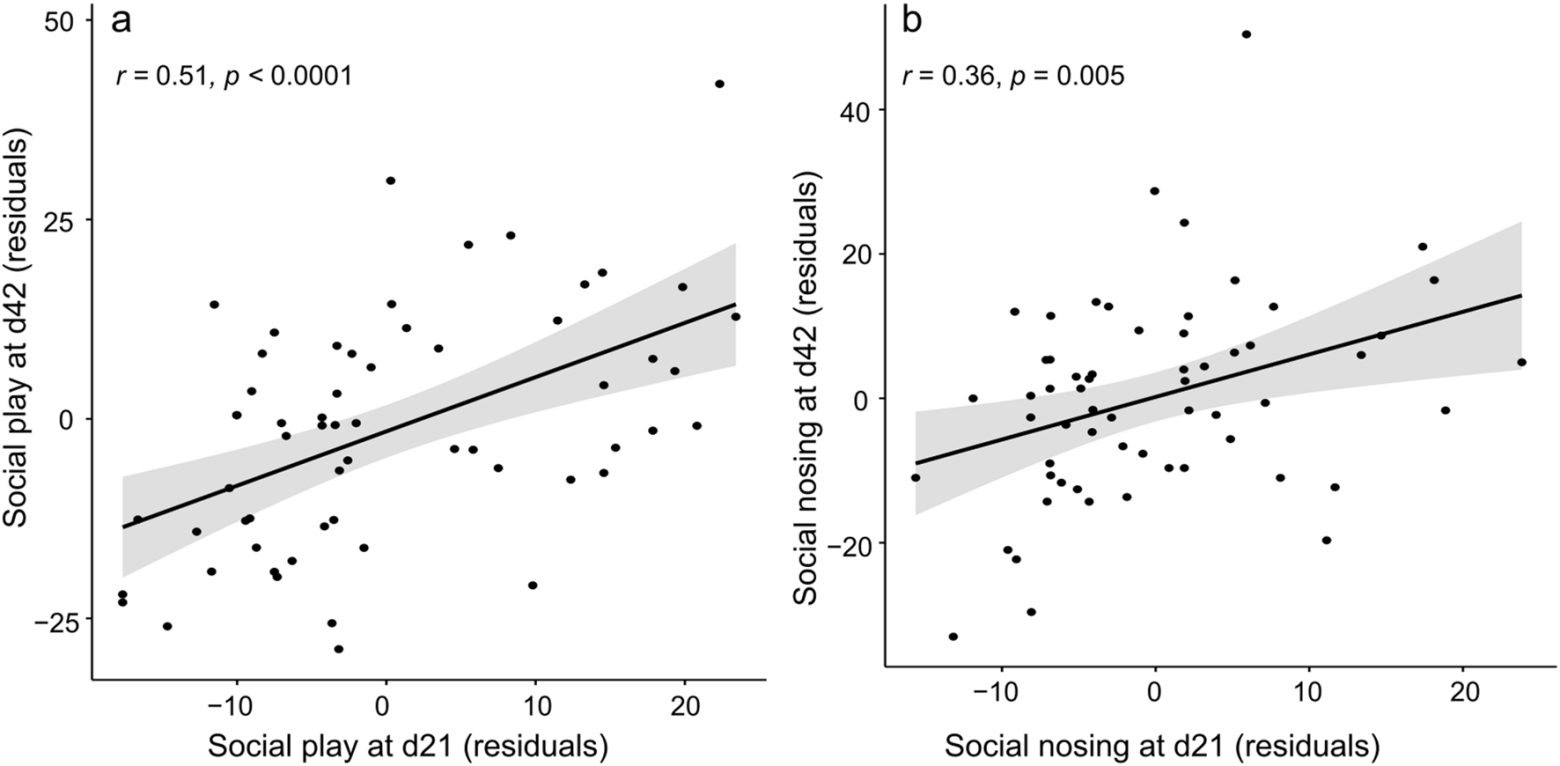
Association between the count of (**a**) social play episodes, and (**b**) social nosing behaviors given at 21 days and 42 days of age. Pearson’s correlation tests were performed on variable residuals obtained from a mixed linear model including the fixed effects of pen and batch. The black line represents the regression line and the grey area represents the 95% confidence interval.

We found positive correlations between social behaviors at 21 and 42 days age. Notably, social play at 21 days were strongly associated to social play at 42 days (Fig. 4a, r = 0.51, *p* < 0.001, *p*_adj_ < 0.001). Moderate positive correlations between 21 and 42 days of age were also found for social nosing (Fig. 4b, r = 0.36, *p* = 0.005, *p*_adj_ = 0.09), agonistic behaviors (*r* = 0.37, *p* = 0.003, *p*_adj_ = 0.07), and other social behaviors given (*r* = 0.34, *p* = 0.009, *p*_adj_ > 0.10). However, the 2 latter correlations were strongly influenced by outliers, and did not resist adjustment for multiple comparisons after outliers were removed (agonistic behaviors given, 1 outlier, *r* = 0.25, *p* = 0.05, *p*_adj_ > 0.10; other social behaviors given, 2 outliers, *r* = 0.25, *p* = 0.05, *p*_adj_ > 0.10).

#### Consistency of individual positions (coordinates) on each PCA dimension

The coordinates of piglets at 21 days of age were positively correlated to the coordinates of piglets at 42 days for PC 1 ‘social exploration & social play’ (*r*_*s*_ = 0.38, *p* = 0.003), but not for PC 2 ‘avoidance & receipt of exploration *vs* aggression’ (*r*_*s*_ = 0.14, *p* > 0.10) or PC 3 ‘receipt of social nosing’ (*r*_*s*_ = 0.04, *p* > 0.10).

#### Stability of piglets’ allocation to each cluster

Of the 60 piglets observed on each day, 48% of all piglets (29/60) remained in the same cluster on both observation days, but the stability of piglets’ allocation to a given cluster varied greatly between clusters. Indeed, 59% (17/29) of piglets classified in the cluster of ‘*low-solicited inactive animals’* on day 21 and 65% (11/17) of piglets classified in the cluster of ‘*socially active animals’* on day 21 remained in the same cluster on day 42, whereas, for the cluster of ‘*highly-solicited avoiders’*, only 7% (1/14) of the piglets remained in the same cluster on both days.

### Associations between social behaviors and 5-HT

Blood levels of 5-HT at 21 days were positively correlated to 5-HT levels at 42 days, but the correlation was weak and did not resist adjustment for multiple comparisons (*r* = 0.29, *p* = 0.03, *p*_adj_ > 0.10). No significant correlations were found between 5-HT and social behaviors at either age (*p* > 0.05 for all), except for a weak negative correlation between 5-HT levels and the number of social play episodes at 21 days (*r* = − 0.25; *p* = 0.04, *p*_adj_ > 0.10).

## Discussion

This study aimed to explore and characterize early social behaviors and social styles in suckling piglets raised with their littermates until 6 weeks of age, and to assess the stability of these social styles over time, and their associations with a variety of individual features, including non-social behaviors, morphological traits, and health-related blood parameters.

### Descriptive analysis of social behaviors in suckling piglets

Descriptive analysis of behaviors showed that (putative) positive social behaviors, namely social nosing and social play, represented a large proportion of all behaviors, whereas the proportion of agonistic behaviors and other social behaviors were low at both ages. Accordingly, low incidence of agonistic interactions compared to non-agonistic social interactions has been reported in stable social groups of juvenile pigs^7,8^. Social nosing is assumed to promote social relationship maintenance and group cohesion and to play a role in the reduction of social tension through positive soothing effects^5,6,11,14,39^. Therefore, the high incidence of social nosing and the low incidence of agonistic behaviors reported in our study likely reflected strong social cohesion and affiliative relationships within litters in a calm and stable social context^10,11,40^, and was likely caused by optimal environmental conditions, including extra space and access to a foraging substrate^7^. Indeed, piglets in our study were housed with littermates, in large enclosure with outdoor access, which enabled them to escape fights, and likely resulted in fewer and less intense agonistic interactions.

Despite stable relative levels of social behaviors over time, the absolute levels of social activities increased with age, notably social play and social nosing. In line with our findings, an increase in time spent interacting with littermates (aggression, nose contact, mounting, and oral manipulation) with age has been reported in pre-weaning piglets^41,42^. More specifically, the greater number of social play episodes with age agrees with prior studies that showed a peak in social play between 2 and 6 weeks of age^43^, followed by a sharp decrease from 5 to 9 weeks of age in a semi-natural or enriched environment with saw bedding^44^. On the contrary, no effect of age was found on the frequency of social play in piglets raised in a poor environment characterized by restricted space^16^, suggesting that enriched environment may favor optimal play development. Social play, including rough-and-tumble play or ‘playfight’, is assumed to help piglets to develop a set of social skills that are important for optimal integration into future hierarchical social groups and for successful fighting and individual recognition^18,19,31^. Like social nosing^2,6,13^, early social play is thought to help maintain group cohesion^2,10^, and may thus be considered a putative positive social behavior important for long-term pig welfare on farms.

Remarkably, although males and females had the same activity levels in general, males were involved in more social interactions than females. Notably, males emitted more agonistic behaviors and other social behaviors, namely mounting and nudging, and were involved in significantly more social play episodes than females. Mounting is often considered a sexual behavior^45^, but is also assumed to play a role in the formation of dominance hierarchy^46^. In line with our findings, entire male pigs have been found to display more agonistic and sexual behaviors, such as mounting, than female pigs^15,45^, although contradictory findings are sometimes reported^46^. Furthermore, in a wide range of farm animals, including horses^47^, lambs^48^, and pigs^19,49,50^, males initiate more juvenile social play than females. Indeed, male pigs have been found to be involved more often in play fighting, pushing and nudging, than females during the pre-weaning period (7 to 27 days of age^50^, 14 to 26 days of age^49^), and after weaning (50 to 68 days of age^19^). These differences in play behaviors support the hypothesis that species in which males and females differ in their later-life social environment show sex differences in their early play behavior, especially fight-related play^47,49,51^. Altogether, our findings thus support the existence of sexual dimorphism in the early expression of social behaviors, notably play fight, mounting or aggression, in young piglets.

### Identification and characterization of individual social styles

The multivariate analyses highlighted the existence of 3 distinct social styles. The largest cluster represented ‘*low-solicited inactive animals’*, which were involved in fewer social interactions than other animals in general. The diametrically opposed cluster was that of ‘*socially-active animals’*, which emitted more social play, social nosing, agonistic behaviors and other social behaviors than other animals. The smallest cluster represented ‘*highly-solicited avoiders’,* which was typically characterized by more avoidance behaviors in response to a high level of social solicitation, as suggested by great counts of received social-nosing, mounting and nudging.

The characterization of social styles differing in their levels of ‘social engagement’ (*i.e.* socially active *vs* inactive animals) agrees with prior studies in cattle which showed that high levels of socio-positive behaviors (social licking) are linked to greater levels of sociality overall, including agonistic behaviors^25^, suggesting that more affiliative or more aggressive animals are more social overall and vice versa. Remarkably, the social styles highlighted in our study were not only characterized by the level of emitted social behaviors, but also by the level of received social contacts (*i.e.* low-solicited *vs* highly-solicited animals). In line with our findings, ‘avoiders animals’ have been characterized by high levels of received aggression, while ‘passive animals’ received high levels of positive contacts in sheep and goats^26–28^. Altogether, these findings suggest that the amount and type of social solicitation received by an individual within a group influence its social responses and highlight the importance of considering both emitted and received interactions when studying social behavior. Furthermore, animals from low dominance status have been shown to have low participation in agonistic or non-agonistic interactions and to avoid conflicts in adult sows^20^, cows^25^, goats^26,27^ and ewes^28^, thus suggesting that dominance status may be an important determinant of social styles or strategies in farm animals. Further studies including assessment of dominance status should thus be considered to confirm whether ‘*highly-solicited avoiders’* or ‘*low-solicited inactive animals*’ are primarily low-ranking animals.

In our study, the 3 distinct social styles were associated with a number of individual features. Notably, ‘*low-solicited inactive animals’* were typically females, whereas ‘*socially-active animals’*, which exhibited high levels of play behavior, nudging and mounting, were predominantly males. Accordingly, males have been found to exhibit more social play, nudging, and mounting than females in suckling^49,50^ and weaned piglets^19^. Furthermore, as mentioned before, mounting in pigs has been described as both a sexual^45^ and a dominance behavior^46^ that is typically displayed by males. Therefore, the social styles highlighted in our study may be partly explained by sexual dimorphism in the expression of social behaviors associated with sex or dominance.

Besides sex, social styles were associated with behavioral and physiological parameters. Indeed, ‘*low-solicited inactive animals’* spent less time standing, and more time lying than animals from other clusters, although this effect was only observed in the sub-groups of females. In line with our findings, inactivity has been associated with little social nosing in weaned piglets^13^, and a passive social style characterized by low incidence of emitted non-agonistic and agonistic interactions and a high proportion of time spent resting (lying) has been reported in goats^26,27^. Remarkably, ‘*low-solicited inactive animals’* also had higher levels of haptoglobin compared to ‘*socially active animals’*. Concentration of serum haptoglobin, a major porcine acute phase protein, rises rapidly in response to infection, and is thus an important marker of health in pigs^33^. A rise in acute phase protein and inflammatory cytokine levels, paired with a decline in social motivation and general activity is assumed to be indicative of ‘sickness behavior’ in pigs, as evidenced by studies using lipopolysaccharide-induced inflammatory challenges^32,52^. The cluster of ‘*low-solicited inactive animals’* may thus be typically composed of (female) animals of bad health status or suffering from sub-clinical infection. Alternatively, accumulated evidence suggest that acute phase proteins may be released not only following an infection, but also in response to physical or psychological stressors^53–55^, and may thus be a promising indicator of stress^13^. Therefore, clusters in our study may partly reflect individual differences in health status, stress level or both on a given day. Remarkably, we reported a relative instability in the piglets’ allocation to a given social cluster over time, with only 48% of piglets belonging to the same clusters at 3 and 6 weeks of age. Together with the association between clusters and inflammation markers, this instability supports the postulate that our clusters may not depict stable sociality traits, but rather transient social mood reflective of the individual’s psychological and physiological health at a specific time point.

The stability of piglets’ allocation to a given cluster varied greatly between clusters, with relatively high levels of stability for ‘*low-solicited inactive animals*’ (59%) and ‘*socially active animals*’ (65%), and very low stability (7%) for the ‘*highly-solicited avoiders’*. The low stability of the cluster of ‘*highly-solicited avoiders*’ may be explained by the “intermediate” position of animals from this cluster in terms of health-related parameters (haptoglobin, growth rate) or behaviors (agonistic behaviors given and received, other social behaviors given, social play), that were major discriminant factors of the two other clusters. Therefore, piglets from the ‘*highly-solicited avoiders*’ cluster may easily move from their cluster to others. Furthermore, when considering position of animals on each PCA axis, we reported positive correlations between ages for PC 1, that represented ‘social engagement’ or ‘sociability’, but not for other PCs which represented other social traits, such as ‘aggressiveness’ and ‘avoidance’, or passive social involvement. While supporting the postulate that different social behaviors reflect distinct dimensions of sociality, as suggested by others^9,56^, our results also suggest that the level of ‘sociability’, may be a stable social trait in young piglets, contrarily to ‘avoidance’ or ‘aggressiveness’. As mentioned earlier, appropriate responses to aggression (avoidance *vs* fight) required for successful conflict resolution later in life is likely acquired through early play fight experience^18,19^. We therefore posit that the development of the ‘avoidance’ and ‘aggressiveness’ traits in young piglets is under the influence of early life experience, thus explaining their instability in young, developing individuals.

Like individual position on the PCA axes, some social behaviors showed relative consistency with age when analyzed independently. Notably, animals involved in the greatest count of social play behaviors at 3 weeks were also the ones playing the most at 6 weeks, maybe because this behavior was highly influenced by the sex of the animals. In line with our findings, counts of play behaviors (jumping, turning and running) and time spent displaying these behaviors during a ‘play session’ have been found to be fairly consistent between 2 consecutive play sessions in 44- to 48-day-old piglets, suggesting that playfulness could be an inherent individual trait^17^. Furthermore, in our study, piglets that were nosing pen mates the most at 3 weeks were also the ones nosing pen mates the most at 6 weeks. As no strong intra-individual associations were found for other social behaviors, we posit that playfulness and social nosing may represent valid and stable indicators of early individual social styles in young piglets raised in enriched environments, and deserve more research effort.

Finally, and contrarily to our expectations, levels of 5-HT in platelet-rich plasma were not associated to any social styles or behaviors in our study, except for a very weak negative association with the count of social play episodes. The motivation to play during daily play sessions has been found to be negatively correlated to the rise in peripheral 5-HT levels before and after a play session in minipigs, suggesting that the more the piglets are willing to play, the less the 5-HT concentrations rise^57^. In line with this finding, our result may support the postulate of a negative association between play behavior and 5-HT levels in pigs. Further investigation in various contexts of play is needed to determine whether peripheral 5-HT level is a promising candidate to characterize social play, and positive affective states, in young piglets.

## Conclusion

To the best of our knowledge, this study is the first to characterize social styles in suckling piglets. Our findings suggest the existence of individual behavioral patterns that seem reflective of transient social styles, and that are behaviorally and physiologically distinct. Although individuals’ allocation to a given social style was not very stable over time, the ‘sociability’ trait and positive social interactions (social nosing and play) appeared to be stable social traits in young developing pigs, contrarily to dominance or sex-related social traits (mounting, avoidance and agonistic behaviors). Altogether, these findings bring innovative insights into early individual social styles in pigs. Further research should consider investigating to what extent piglets with divergent early social styles differ in their ability to adapt to weaning, a stressful social challenge on pig farms.

## Methods

### Ethical notes

The project was conducted at the French National Institute INRAE organic pig farm Porganic (Rouillé, France) from May to October 2020 in compliance with the current ethical standards of the European Community (Directive 2010/63/EU). The experimental procedures used in this study were approved by the Regional Ethics Committee in Animal Experiments of Poitou Charente (n°084, December 18, 2019) and by the French Ministry of Higher Education and Research (2019071611422718Apafis21892). This research was done in compliance with the Animal Research: Reporting of In Vivo Experiments (ARRIVE) guidelines and regulations (https://arriveguidelines.org).

### Animals and housing

Piglets (Large White × Piétrain) from 16 litters (15 ± 0.6 piglets born alive, [range: 10–20]; male:female ratio = 1.4) were studied in 2 consecutive cohorts, with 6-week intervals between cohorts and 8 litters per cohort. All piglets were kept with the sows and housed in 10 m^2^ individual farrowing pens, located within the same maternity unit, from birth until 48 days of age. From 11 days of age, sows and their piglets had free access to a 6.25-m^2^ outdoor area. Sows were loose and pens were equipped with a heated nest only accessible to the piglets, and had a straw-bedding floor. Fresh straw was added daily. Male piglets were not castrated, teeth were not clipped and tails were kept intact. If needed, cross-fostering was done in the first 3 days of age to standardize litter size. Piglets received daily access to solid feed from 20 days of age onwards, and were weaned at 48 days of age.

Piglets did not receive iron injection because the experiment used piglets from a project that explored whether daily access to soil or peat during the suckling period could be an applicable alternative strategy to the early iron injection to prevent risks of anemia in suckling piglets raised in organic farms (European ERA-Net project). Therefore, at about 4 days of age, litters were randomly allocated to 1 of 2 treatments, with 4 litters per treatment per cohort, thus yielding a total of 8 litters per treatment in total. Piglets received daily access to a small amount of sterilized soil or peat from 4 to 48 days of age. Soil and peat were distributed daily at 9:00 in a small circular feeder located in the nest and not accessible to the sow. Daily rations of soil and peat per pen were 150 g from 4 to 12 days of age, 200 g from 13 to 26 days of age, and 250 g from 27 to 47 days of age.

### Measurements

Per litter, 6 experimental piglets (3 males and 3 females) of medium birthweight (1.51 ± 0.03 kg, [0.82–2.32 kg]) were selected for blood sampling and behavioral observations, resulting in 96 experimental piglets in total. Adopted piglets and piglets with health problems (diseases or lameness) were excluded from the selection. If a piglet died between 2 observation days, it was replaced by a piglet from the same sex and litter.

#### Physiological measures

At 20 and 41 days of age, all piglets were weighed, and blood was collected from the 96 experimental piglets. The sampling procedure started at the same time (~ 09:00 AM) on both days, and blood from the piglets was collected in a random order. At 20 days of age, pigs were maintained on their back on a table by a trained experimenter, and at 41 days of age, pigs were restrained with a wire metal nose snare. The duration of the blood sampling procedure was measured, and took less than 2 min per pig, thus limiting any potential effect of stress on physiological and behavioral measures. Blood was collected into one 5-mL BD Vacutainer EDTA and one heparinized tube by jugular venipuncture and were kept on ice until processing.

Within one hour after sampling, platelets counts in whole blood (10^9^ platelets/L) were measured in EDTA samples with a hematology automated cell counter for pigs (MS9; Melet Schloesing Laboratories, Osny, France). Whole blood EDTA samples were then centrifuged at 200 × g at room temperature for 10 min to obtain platelet-rich plasma (PRP). Samples of 200 µL of extracted PRP were added with 800 µL saline and centrifuged at 4500 × g for 10 min at 4 °C. The supernatants were retrieved, the pellets were resuspended in 200 µL of distilled water and PRP samples were stored at − 80 °C until 5-HT analyses. The 5-HT concentrations were determined using an ultra-performance liquid chromatography (UPLC) apparatus (Waters Acquity Ultra Performance LC, Waters, Milford, MA, USA) coupled to 2 detectors (Acquity Tunable UV detector and Mass SQD detector; Waters, Milford, MA, USA) after derivatization of samples using the AccQ Tag Ultra method (MassTrak AAA; Waters, Milford, MA, USA). Norvaline (Sigma-Aldrich, Saint Quentin Fallavier, France) was used as an internal standard. Concentrations of 5-HT were expressed in μmol/mL of PRP, and inter-assay CV was 11%.

Heparinized blood samples were centrifuged at 1800 × g for 10 min at 4 °C. Plasma was collected and stored at − 20 °C until analyses of inflammatory (haptoglobin) and oxidative stress markers (hydrogen peroxide and plasma antioxidant capacity). Haptoglobin (Tridelta Development Ltd, Maynooth, Ireland), and hydrogen peroxides generated by the peroxidation of lipids, proteins or nucleic acids (diacron Reactive Oxygen Metabolites , dROM kit, H&D srl, Parma, Italy) were assayed using commercial kits. The ferric reducing ability of plasma (FRAP) was assayed using a home-made assay, as previously described^58^. All measurements were performed in duplicates using a multianalyzer apparatus (Konelab 20i, ThermoFisher Scientific, Courtaboeuf, France). The minimum concentration detectable for haptoglobin was 0.033 mg/mL, and the intra- and inter-assay coefficients of variation (CV) were 7 and 24%, respectively. Concentrations of dROM were expressed in CARRU (Carratelli Unit, 1 CARRU = 0.08 mg H_2_O_2_/100 mL of sample) and intra- and inter-assay CV were 6 and 8%, respectively. FRAP was expressed as molar Trolox equivalents/L.

#### Behavioral measures

Immediately after blood sampling, the experimental piglets were individually marked with a symbol sprayed on their back, and the next day, video were continuously recorded for the analysis of social behavior from 06:30 to 19:30 at 21 and 42 days of age. Social behaviors and non-social behavioral activities were scored during four 2-h sessions of observation (8:00–10:00, 11:00–13:00, 14:00–16:00, and 17:00–19:00) on both days.

Social behaviors were scored using the all-occurrence behavioral sampling method. A total of 5 categories of social behaviors were defined (Supplemental Table S1): social nosing behaviors (nosing snout, head or body), agonistic behaviors (head and shoulder knocks, aggressive bites), other social behaviors (mounting and nudging), avoidance behaviors (turning head or moving away from an interaction initiated by a pen mate, refusing play invite), and social play (play invite, mutual play-fight, chasing, climbing or pushing^49^). For social nosing, agonistic and other social behaviors, both the social interactions given and received by focal animals were scored, resulting in 8 behavioral categories in total.

Non-social behavioral activities were scored using a 5-min scan sampling during the four 2-h sessions of observation on both days, resulting in 96 samples per pig per day. A total of 4 categories of activities were scored: standing/kneeling active (exploring, playing, walking or running), lying/sitting active or inactive (exploring, resting), ingestive behaviors (drinking, feeding), suckling or massaging the udder. Supplemental Table S2 shows the detailed behavioral repertoire.

### Statistical analyses

Data analysis was conducted using the statistical R version 4.0.0^59^ with the packages ‘lme4’ version 1.1-26, ‘FactoMineR’ version 2.3, ‘factorextra’ version 1.0.7, and ‘ggplot2’ version 3.3.1.

#### Descriptive analysis of behavioral data

Of the 96 experimental pigs from 16 litters, 4 litters were excluded (2 due to high mortality between 21 and 42 days of age, 1 due to piglets being weaned before 42 days of age, and 1 because the video recordings failed), thus resulting in a sample of 12 litters (21 days: 11 ± 0.5 piglets per litter; 42 days: 10 ± 0.6 piglets per litter). Furthermore, 6 piglets (4 at 21 days and 3 at 42 days of age) could not be identified on the video recordings (marking was erased) and were thus excluded from the observations, resulting in a final sample of 68 piglets at 21 days and 69 piglets at 42 days of age.

Behavioral data were analyzed with linear mixed models including the fixed effects of day, sex and treatment, and the random effect of pen nested within batch. Interactions between fixed effects were not significant and were, therefore, not included in the final model. Model residuals were visually inspected for normality and homoscedasticity, and if the residuals did not meet the assumptions for normal distribution and equality of variances, response variables were transformed (square-root transformation for count data and arcsin transformation for proportion data). Data are presented as means ± SEM.

#### Identification of early social styles

A global principal component analysis (PCA) was done to analyze the correlational structure between the 8 social behavioral classes at 21 and 42 days of age. Only the 60 animals (29 females and 31 males) that were observed at both 21 and 42 days of age were included in the PCA. The behavioral items included as active variables in the PCA were the count of agonistic behaviors given and received, the count of social nosing behaviors given and received, the count of other social behaviors given and received, the count of social play episodes and avoidance behaviors. The total number of social interactions was added as a supplementary quantitative variable, and sex, day and treatment were added as qualitative supplementary variables. All active variables were subjected to a linear model with pen and batch as fixed effect to obtain residuals used for the PCA. The criteria for extracting principal components were an eigenvalue > 1 and a minimum cumulative variance of 50%. The extracted principal components were described with the variable residuals with loadings > 0.50 or < ‒0.50. A hierarchical clustering on principal components (HCPC) was performed on the principal components extracted from the PCA to identify clustered groups of pigs based on social behaviors. The Euclidean distance was used between individuals and the Ward’s criterion was applied as clustering method^60^.

#### Characterization of clusters with non-social individual parameters

Statistical tests were computed using the HCPC function of the ‘FactorMineR’ package to identify the principal components and variables used in the multivariate analyses that characterized the clusters. Principal components and the supplementary quantitative variable (total number of social interactions) characterizing the clusters were identified using *v*-tests – a *v-*test greater than 1.96 or less than − 1.96 corresponds to a *p* value less than 0.05, and the sign of the *v*-test indicates if the mean of the cluster is lower or greater than the overall mean of all clusters^60^. Qualitative variables (sex, day, treatment) characterizing the clusters were identified using, first, a chi-square test (χ^2^) and, then, hypergeometric tests to determine which clusters were characterized by each category of the qualitative variables^60,61^. Because clusters were not significantly associated with treatment in these tests (χ^2^, *p* > 010), treatment effects were not discussed further.

The association between clusters and the quantitative variables that were not used for the PCA and HCPC (non-social behavioral traits, production traits and blood parameters) was assessed with one-way ANOVA using mixed linear models including the fixed effects of cluster, sex, treatment and their two-by-two interactions, and the random effects of day and pen nested within batch. Treatment (daily access to soil or peat) or its interaction with cluster or sex had no effect on any variables, and was thus excluded from the final model. Model residuals were visually inspected for normality and homoscedasticity, and if these assumptions were not met, models were performed on transformed variables (square-root transformation for count data, and arcsin square-root transformation for proportion data). Data are presented as LS-means ± S.E.M., unless stated otherwise.

#### Stability of social-related traits

A series of correlation tests were conducted to assess the constancy of social-related parameters (social behaviors given, blood levels of 5-HT, and individual positions on PCA axes) over time. Raw variables (social behaviors and 5-HT levels) were first subjected to a linear model with pen and batch as fixed effects to obtain residuals, as previously described by Ursinus et al*.*^38^. Because model residuals were normally distributed, parametric Pearson’s correlation tests were conducted on the variable residuals. Both *p* values and adjusted *p* values corrected for multiple comparisons using the Holm method are shown. The stability of individual coordinates on each of the 3 extracted principal components was assessed with non-parametric Spearman’s correlation tests.

## Supplementary Information

Supplementary Information.
