# Supplementary material for: Exploration of early social behaviors and social styles in relation to individual characteristics in suckling piglets
Source: Sci Rep. 2022 Feb 10;12:2318. doi: 10.1038/s41598-022-06354-w (PMC8831595; doi:10.1038/s41598-022-06354-w)
Supplement: Supplementary file 1 — Supplementary Information. [file 41598_2022_6354_MOESM1_ESM.docx]

**Supplemental Table S1.** Behavioral repertoire of social behaviors in suckling piglets

| Behavior | Description |
| --- | --- |
| Social nosing behaviors | |
| Nosing nose | The pig approaches or turns its head toward another pen mate and touches the snout of the pen mate with its snout. If it is difficult to identify the initiator pigs, both pigs are identified as emitters of 'nosing nose'. |
| Nosing head | The pig touches, gently rubs or licks the head (including ear) of a pen mate with its snout, including licking and nibbling hairs or eyelashes. Can result in, but does not include biting. |
| Nosing body | The pig touches, gently rubs or licks the body (including legs, neck and tail) of a pen mate with its snout, including licking and nibbling hairs. Can result in, but does not included tail biting. |
| Agonistic behaviors | |
| Aggressing | The pig gives a head or shoulder knock, i.e. strikes another pig with significant force, or aggressively bite any part of the body of a pen mate. Can result in, but does not include active reciprocal fight. |
| Other social behaviors | |
| Nudging | The pig does a single gentle push or up and down movement with its snout on the body of a pen mate, including legs when pig is lying down but excluding the belly. Usually occurs in bouts of behaviors in quick succession. |
| Mounting | The pig stands on hind legs while having front legs on other pig's body. |
| Social play behaviors | |
| Play-fight | The pig gives frontal head or shoulder knock with minimal, moderate or substantial force to another animal to invite it to play fight. Mutual ramming or pushing, with or without non-aggressive biting. |
| Rough and tumble play | Chasing: The pig run and chase another or other pigs intensely with rapid changes in direction. May be associated with barkings and gently nudging of pen mates. Pushing: The pig drives its head or shoulders with minimal, moderate or substantial force at a target piglet, excluding frontal play invite. May result in the target to lose balance and fall over. Climbing: The pig climbs or attempts to do so from the side or front of another pig. Play behavior is only scored once per playing bout. A playing bout is finished when the focal pig stops running, chasing or pushing other pig for at least 10 sec or engages in another activity. Play is not associated with delivery or receipt of aggression and does not include pushing past other pigs restricting passage during locomotion, suckling at the udder or joining a resting pile of pigs. |
| Avoidance behavior | |
| Avoiding contact | The target pig actively avoids the contact (nosing, nudging, biting and aggression) by either turning its head or body away or moving away with no further reaction. |
| Avoiding play | The pig actively avoids play invite by not responding to the invitation, by turning its body away or by moving away with no further reaction. |

**Supplemental Table S2.** Behavioral repertoire of non-social behavioral activities in suckling piglets

| **Behavior** | **Description** |
| --- | --- |
| Standing or kneeling | The pig is standing on its four legs or kneeling on its front knees. It may explore the environment or its pen mates, or walk or run in the pen |
| Lying or sitting | The pig is lying with its eyes closed (resting) or opened or is sitting on its back rear. The animal can be inactive or exploring the substrate or pen mates. |
| At the feeder | The pig is exploring or eating soil or peat from the feeder. |
| At the drinking trough | The pig is exploring or drinking water from the drinking trough. |
| At the udder | The pig is actively suckling or massaging the udder with repetitive up-and-down snout movements. Animals that are asleep at the udder or lying inactive with or without a teat in their mouth are scored as “lying”. |
